# Comparative safety and efficacy of anti-PD-1 monotherapy, chemotherapy alone, and their combination therapy in advanced nasopharyngeal carcinoma: findings from recent advances in landmark trials

**DOI:** 10.1186/s40425-019-0636-7

**Published:** 2019-06-25

**Authors:** Jia-Wei Lv, Jun-Yan Li, Lin-Na Luo, Zi-Xian Wang, Yu-Pei Chen

**Affiliations:** 1 Department of Radiation Oncology, Sun Yat-sen University Cancer Center, State Key Laboratory of Oncology in South China, Collaborative Innovation Center for Cancer Medicine, Guangdong Key Laboratory of Nasopharyngeal Carcinoma Diagnosis and Therapy, 651 Dongfeng Road East, Guangzhou, 510060 People’s Republic of China; 2 Department of Medical Oncology, Sun Yat-sen University Cancer Center, State Key Laboratory of Oncology in South China, Collaborative Innovation Center for Cancer Medicine, Guangzhou, People’s Republic of China

**Keywords:** Nasopharyngeal carcinoma, Anti-PD-1, Chemotherapy, Combination therapy, Safety profiles, Efficacy, Predictive biomarker

## Abstract

Recent phase 1–2 trials reported manageable safety profiles and promising antitumor activities of anti-PD-1 drugs (pembrolizumab, nivolumab, camrelizumab and JS001) with/without chemotherapy in recurrent/metastatic nasopharyngeal carcinoma (RM-NPC), however head-to-head comparison among these regimens is lacking. We aimed to comprehensively compare the efficacy and safety of different anti-PD-1 drugs, standard chemotherapy, and their combination therapy in RM-NPC. Adverse event (AE) and objective response rate (ORR) were assessed. The pooled incidence rates of grade 1–5/3–5 AEs were 74.1%/29.6, 54.2%/17.4, 92.3%/24.5, 96.8%/16.1, 91.2%/42.8, and 100%/87.9% for pembrolizumab, nivolumab, JS001, camrelizumab, chemotherapy and camrelizumab+chemotherapy, respectively, which suggested that nivolumab and pembrolizumab exhibited the optimal safety regarding grade 1–5 AEs whereas camrelizumab and nivolumab regarding grade 3–5 AEs. As second- or later-line therapy, ORR was higher with camrelizumab (34.1%), followed by pembrolizumab (26.3%), JS001 (23.3%), and nivolumab (19.0%); whereas ORR with first-line nivolumab reached 40%. Additionally, first-line camrelizumab+chemotherapy achieved a dramatically higher ORR than that with chemotherapy alone (90.9% vs. 64.1%). Pooled ORR was 28.4 and 17.4% for PD-L1–positive and PD-L1–negative patients, respectively (*P* = 0.11). Here, we represent preliminary evidence for the comparative safety and efficacy of existing anti-PD-1 agents with/without chemotherapy in RM-NPC, which indicated that camrelizumab has the least toxicity profile and merits future investigation. Our findings might provide insights into the future design of immunotherapy trials in RM-NPC.

## Background

Nasopharyngeal carcinoma (NPC) is one of the most common head and neck cancers in Southeast Asia and North Africa. The age-standardized incidence ranges from 20 to 50 per 100,000 males in southern China to 0.5 per 100,000 in white populations [1]. Recently, the first phase 3 trial in recurrent or metastatic NPC (RM-NPC), the landmark GEM20110714 study, has established gemcitabine plus cisplatin (GP) regimen as the standard first-line treatment [2]. However, no consensus has been reached beyond the first-line setting, in which the prognosis is extremely poor.

Endemic NPC is etiologically associated with Epstein-Barr virus infection. This virus-associated cancer represents the archetypal “inflamed tumor,” which exhibits a dense lymphocytic infiltrate and increased programmed death-ligand 1 (PD-L1) expression [3, 4]. These features make immunotherapy a promising treatment option for NPC patients. Recently in 2017, the landmark KEYNOTE-028 trial firstly reported promising antitumor activities and safety profiles of pembrolizumab in previously treated RM-NPC [5]. Subsequently, five additional phase 1–2 trials evaluating anti-PD-1 antibodies in RM-NPC were reported [6–9]. The NCI-9742 [6] and CheckMate-385 [7] trials demonstrated a manageable safety profile and clinical activity of nivolumab in multiply pretreated and/or treatment-naive RM-NPC patients. Fang and colleagues [8] reported that camrelizumab monotherapy was a well-tolerated and potentially effective treatment option for previously treated RM-NPC. They further reported that the combination of camrelizumab plus chemotherapy of GP regimen has a manageable toxicity profile and promising preliminary antitumor activity in treatment-naive RM-NPC [8]. Another latest trial, the JS001 study, reported in the European Society for Medical Oncology (ESMO) 2018 conference demonstrated the clinical activity of JS001 in multiply pretreated RM-NPC [9]. However, to date there is no head-to-head comparison of different anti-PD-1 drugs, standard first-line GP chemotherapy, and their combination therapy in RM-NPC. Therefore, we initiated this study to comprehensively compare the safety and efficacy of the abovementioned trials, and explore the optimal therapeutic regimens of anti-PD-1 approach in RM-NPC. We hypothesized that the efficacy and safety profiles differed across different anti-PD-1-based regimens.

## Methods

The abovementioned anti-PD-1 trials were included in the analysis with GP arm from GEM20110714 trial as chemotherapy control [2]. The major assessed outcomes were adverse event (AE) and objective response rate (ORR). AE and ORR data were pooled up per regimen and described in percentage. The comparative incidences of AE between different regimens were evaluated by the odds ratio (OR) and corresponding 95% confidence interval (CI) using Fisher’s exact test. OR > 1 stands for fewer AEs. When AE rate in any comparative arm equaled 100% or 0%, the Haldane-Anscombe correction was adopted to evaluate OR and its 95% CI [10]. Given that ORR of PD-1 blockade may differ according to treatment lines (first-line vs. >1st line), we also evaluated the anti-PD-1 drugs per treatment setting and considered them as independent comparative groups when data was available. Given the evidence that high PD-L1 expression tended to be associated with favorable responses to PD-1 blockade in NPC [6], we further evaluated the pooled ORR of anti-PD-1 therapies stratified by PD-L1 positivity. Statistical analyses were performed using R version 3.5.1 (http://www.r-project.org). A two-tailed *P* < 0.05 was considered statistically significant.

## Results

### Safety profile of different regimens

**Fig. 1:**
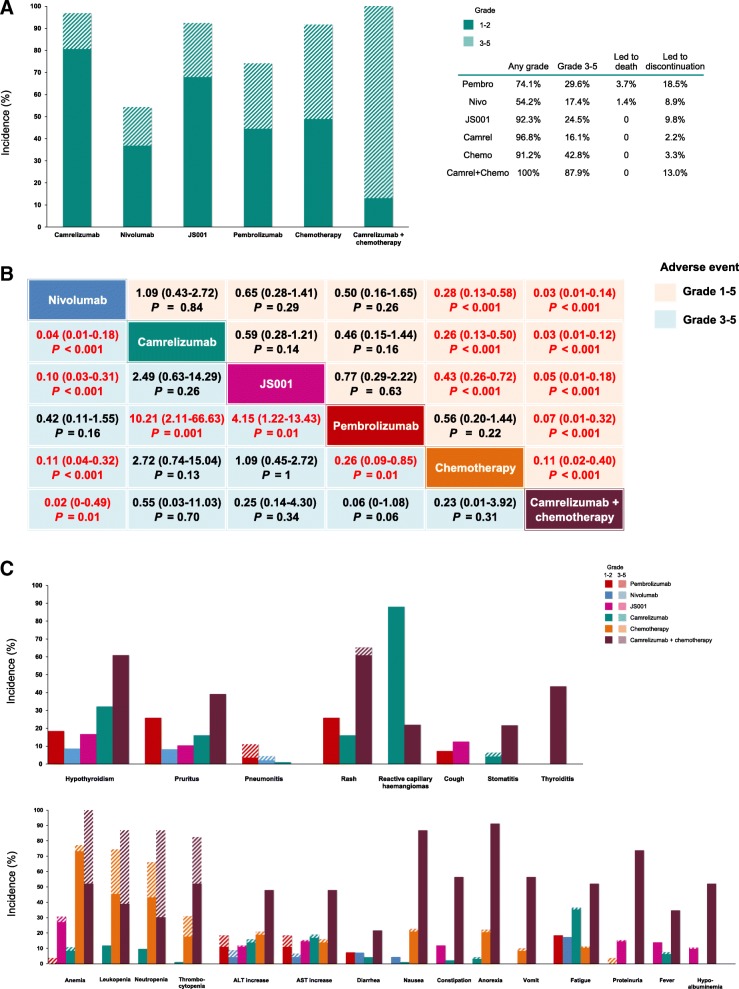
Safety profiles of anti-PD-1 monotherapy, chemotherapy alone, or their combination in advanced nasopharyngeal carcinoma. **a** Bar plot depicts the incidence rates of grade 1–5 adverse events (divided into grade 1–2 and 3–5) in pembrolizumab, nivolumab, JS001, camrelizumab, chemotherapy, and camrelizumab plus chemotherapy. The rates of deaths and discontinuation rates due to adverse events are also presented. **b** Indirect comparisons of grade 1–5 and 3–5 adverse events in different regimens. The pooled odds ratios and 95% confidence intervals indicate the result of the top regimen versus the bottom regimen. Each cell contains the pooled odds ratios and 95% confidence intervals; significant results are indicated in red. **c** Bar plot depicts the toxicity spectra based on each of the specific adverse event. The upper section shows the incidence rates of immune-related adverse events and the lower section shows the incidence rate of other common class-specific adverse events. The incidence rates of pneumonitis in camrelizumab plus chemotherapy, and thyroiditis, proteinuria, hypoalbuminemia and vomit in camrelizumab were reported to be zero. The grade 1–2 adverse events of anemia and proteinuria were not reported for pembrolizumab

Table 1 summarizes the characteristics of included trials. The median sample size for anti-PD-1 monotherapy was 45 (range, 24–143), sample sizes for combination therapy and GP chemotherapy were 23 and 181, respectively. Four of the seven (57.1%) trials investigated anti-PD-1 therapy in pretreated RM-NPC, 2/7(28.5%) trials investigated treatment-naive RM-NPC, while one trial (CheckMate-385) investigated patients receiving ≤2 prior systemic therapies. Figure 1 shows the comparison of safety profiles of anti-PD-1 monotherapy, chemotherapy alone, and their combination. The pooled incidence rates of grade 1–5/3–5 AEs were 74.1%/29.6, 54.2%/17.4, 92.3%/24.5, 96.8%/16.1, 91.2%/42.8, and 100%/87.9% for pembrolizumab, nivolumab, JS001, camrelizumab, chemotherapy, and camrelizumab+chemotherapy, respectively (Fig. 1a). The incidence rate of grade 1–5 AEs was lowest with nivolumab monotherapy, while grade 3–5 AEs was lowest with single-agent camrelizumab. Treatment-related deaths were reported in patients receiving pembrolizumab (sepsis, *n* = 1) and nivolumab (pulmonary tuberculosis, *n* = 1) (Fig. 1a). Treatment discontinuation due to AEs was most commonly recorded in pembrolizumab (18.5%), followed by camrelizumab+chemotheray (13.0%) and JS001 (9.8%), while lowest in camrelizumab (2.2%) (Fig. 1a). Fisher’s exact test indicated a noticeably lower risk of grade 1–5 AEs favoring nivolumab and pembrolizumab over other regimens, while nivolumab and camrelizumab demonstrated superior safety ranking to other regimens for grade 3–5 AEs (Fig. 1b). Generally, risks of grade 1–5 and 3–5 AEs of anti-PD-1 agents were lower than those of chemotherapy alone, while their combination therapy shared the highest incidence of grade 1–5 and 3–5 AEs (Fig. 1b).

**Table 1:** Summary of trial- and patient-level characteristics and clinical endpoints of included trials

Items	KEYNOTE-028	NCI-9742	CheckMate-358	JS001	SHR-1210 (monotherapy)	GEM20110714^a^	SHR-1210 (combination)
Trial-level characteristics							
Region	Taiwan	International-collaborated	International-collaborated	Mainland China	Mainland China	Mainland China	Mainland China
Inclusion period	2014–2016	2015–2016	2015–2017	2016–2018	2016–2017	2012–2015	2017–2017
Phase	1	2	1/2	2	1	3	1
Key eligibility criteria	Recurrent/metastatic NPC; Failure on prior standard therapy; PD-L1 expression ≥1%	Recurrent/metastatic NPC; Failure on at least one prior line of platinum-based chemotherapy	Recurrent/metastatic NPC; ≤ 2 prior systemic therapies	Recurrent/metastatic NPC; Failure on at least one prior line of platinum-based chemotherapy	Recurrent/metastatic NPC; Failure on at least one prior line of platinum-based chemotherapy	Treatment-naive recurrent/metastatic NPC	Treatment-naive recurrent/metastatic NPC
Experimental regimen	Anti-PD-1: Pembrolizumab 10 mg/kg q2wks up to 2 years or until disease progression or unacceptable toxicity	Anti-PD-1: Nivolumab 3 mg/kg q2wks on a 4-week cycle until disease progression	Anti-PD-1: Nivolumab 240 mg/kg q2wks until disease progression	Anti-PD-1: JS001 3 mg/kg q2wks until disease progression or unacceptable toxicity	Anti-PD-1: Camrelizumab at escalating doses of 1, 3 and 10 mg/kg, and a bridging dose of 200 mg per dose q2wks until unacceptable toxicity	Chemotherapy: Gemcitabine 1 g/m^2^ (days 1 and 8), and cisplatin 80 mg/m^2^ (day 1) q3wks for six cycles	Anti-PD-1 + chemotherapy: Camrelizumab 200 mg (day 1), gemcitabine 1 g/m^2^ (days 1 and 8), cisplatin 80 mg/m^2^ (day 1) q3wks for six cycles followed by camrelizumab 200 mg maintenance q3wks
Sample size	27	45	24	143	93	181	23
Patient-level characteristics							
Age, median (range), years	52 (18–68)	57 (37–76)	51 (NR)	46 (24–71)	45 (38–52)	47 (39–55)	44 (34–51)
Sex, male, *n* (%)	21/27 (77.8)	35/45 (77.8)	21/24 (88%)	121/143 (84.6)	75/93 (81)	148/181 (83.1)	17/23 (74)
PD-L1 expression^b^							
< 1%, *n* (%)	0	24/42 (57.1)	–	76/136 (55.9)	–	–	–
≥ 1%, *n* (%)	27/27 (100)	18/42 (42.9)	–	60/136 (44.1)	–	–	–
Clinical endpoints							
Median follow-up, months	20.0	12.5	26.0	NR	9.9	22.0	10.2
ORR (%)	26.3	20.5	20.8	23.2	34.1	64.1	90.9
OS							
Median (months)	16.5	17.1	NR	NR	NR	29.1	NR
1-year rate (%)	63.0	59.0	NR	NR	NR	83.2	NR
PFS							
Median (months)	6.5	2.8	2.4	NR	9.9	7.0	10.2
1-year rate (%)	33.4	19.3	NR	NR	27.1	19.6	61.4
All grade AEs (%)	74.1	NR	54.2	92.3	96.8	91.7	100
Grade 3–5 AEs (%)	29.6	22.2	8.3	24.5	16.1	42.8	87.0

*Abbreviations*: *AEs* adverse events, *EBV* Epstein-Barr virus, *GP* Gemcitabine and Cisplatin, *NPC* nasopharyngeal carcinoma, *NR* not reported, *ORR* objective response rate, *OS* overall survival, *q2/3wks* every 2/3 weeks, *PFS* progression-free survival, *PD-1* programmed death-1, *PD-L1* programmed death-ligand 1

^a^ Only paients from the GP arm was included, as it proved that GP regimen significantly prolonged PFS in patients with recurrent or metastatic NPC compared to standard fluorouracil plus cisplatin regimen, and currently GP is used as the first-line therapy for recurrent/metastatic diseases

^b^ Methods of PD-L1 expression evaluation, KEYNOTE-407 trial: The PD-L1 expression was assessed baseline on an archived formalin-fixed, paraffin-embedded tumor sample or a newly obtained biopsy sample using a laboratory-developed prototype immunohistochemical assay (QualTek Molecular Laboratories, Goleta, CA). PD-L1 positivity was defined as membranous staining on 1% or more of a modified proportion score or interface pattern. NCI-9742 trial: The PD-L1 expression was assessed based on paraffin-embedded NPC tumors using the immunohistochemical analysis of PD-L1 (anti-human PD-L1 antibody, clone 22C3, PD-L1 IHC 22C3; pharmDx assay; Agilent Technologies, Santa Clara, CA). PD-L1 expression in tumor cells and immune cells was scored as the percentage of tumor cells and immune cells with membranous straining, respectively. JS001 trial: The PD-L1 expression was assessed based on paraffin-embedded NPC tumors using the immunohistochemical analysis of PD-L1 (anti-human PD-L1 antibody, clone SP142; Spring BioScience, Pleasanton, CA)

To profile the toxicity spectra of different regimens, we further evaluated the incidence of immune-related and other class-specific common AEs (Fig. 1c). Immune-related AEs among different anti-PD-1 drugs included hypothyroidism (range, 8.7–32.3%), pruritus (8.3–16.1%), and rash (16.1–25.9%); camrelizumab was reported to have a notably high incidence of reactive capillary haemangiomas (88.0%). Majority of immune-related AEs were mild and moderate (grade 1–2). Grade 3–5 immune-related AEs included pneumonitis (7.4% in pembrolizumab and 2.2% in nivolumab), rash (4.3% in camrelizumab plus chemotherapy), and stomatitis (2.2% in camrezumab alone) (Fig. 1c). In terms of other common AEs, elevated alanine aminotransferase or aspartate aminotransferase, fatigue, and anemia were observed (Fig. 1c). Of note, the incidence of immune-related and other common AEs increased substantially in camrelizumab+chemotherapy, compared to camrelizumab or chemotherapy alone: common grade 1–5 AEs included anemia (100%; grade 3–5, 47.8%), anorexia (91.3%), neutropenia (87.0%; grade 3–5, 56.6%), leukopenia (87.0%; grade 3–5, 47.8%), nausea (87.0%), thrombocytopenia (82.6%; grade 3–5, 30.4%), proteinuria (73.9%), rash (65.2%; grade 3–5, 4.3%), hypothyroidism (60.9%); the incidences of thyroiditis (43.5%) and pruritus (39.1%) were also relatively higher (Fig. 1c).

### Efficacy of different regimens

**Fig. 2:**
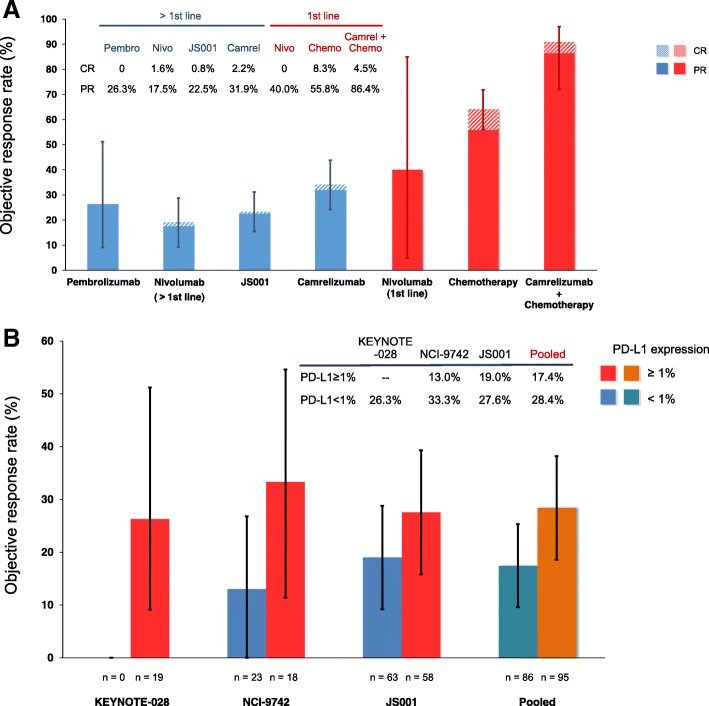
Efficacy of anti-PD-1 monotherapy, chemotherapy alone, or their combination therapy in advanced nasopharyngeal carcinoma. **a** Bar plot shows the proportion of patients with response to pembrolizumab, nivolumab (first-line and > 1st line), JS001, camrelizumab, chemotherapy, and camrelizumab plus chemotherapy. **b** Bar plot depicts objective response rates of anti-PD-1 therapies according to the level of programmed death-ligand 1 (PD-L1) expression; data were available from the three trials (KEYNOTE-028, NCI-9742, and JS001) and their pooled analysis. KEYNOTE-028 trial only enrolled patients with PD-L1-positive tumors. Error bars reflect 95% confidence intervals of objective response rates. CR = complete response, PR = partial response

Figure 2a presents the efficacy of different regimens. The ORR of anti-PD-1 monotherapy used as >1st line therapy ranged 19.0–34.1%, relatively higher in camrelizumab (34.1%), followed by pembrolizumab (26.3%), JS001 (23.3%), and nivolumab (19.0%). Intriguingly, when nivolumab was used as first-line therapy, its ORR increased to 40.0% (Fig. 2a). Camrelizumab+chemotherapy combination treatment in first-line therapy dramatically increased the ORR from 64.1% (chemotherapy alone) to 90.9% (Fig. 2a). It was noteworthy that similar complete response (CR) rates between GP chemotherapy (8.3%) and anti-PD-1 + chemotherapy (4.5%) were observed, though the partial response (PR) rate of anti-PD-1 + chemotherapy (86.4%) was substantially higher than that of GP chemotherapy (55.8%). Pooled ORR for PD-L1–positive patients was 28.4% versus 17.4% of those with PD-L1–negative tumors (*P* = 0.11) (Fig. 2b).

## Discussion

This is the first report that compares the safety and efficacy of different anti-PD-1 drugs with/without chemotherapy in RM-NPC, which provides preliminary evidence and integrative insights into the future design and implementation of immune clinical trials in NPC. The general safety of nivolumab and pembrolizumab ranked high, while the incidences of grade 3–5 AEs were relatively low in camrelizumab and nivolumab. Integrating with the specific toxicity spectra of each drug, we postulate that camrelizumab has the least toxicity profile; the high incidence of all-grade AEs may be attributed to reactive capillary hemangiomas, which is generally unthreatening and self-resolved [8]. In general, anti-PD-1 therapy was safer than standard chemotherapy; however, once it was combined with chemotherapy, the incidence of grade 3–5 AEs and AE-related discontinue rate doubled over chemotherapy alone, which suggests potentially synergized toxicity. This is in accordance with the safety profiles in the KEYNOTE-048 trial investigating anti-PD-1 + chemotherapy versus chemotherapy alone in non-nasopharynx head and neck cancer [11].

The response rate with anti-PD-1 monotherapy for pretreated RM-NPC approximated 20–30%, whereas ORR increased to 40% in treatment-naive patients. Additionally, the incidences of AEs with anti-PD-1 regimen were generally lower than those with chemotherapy. Though the sample size in first-line anti-PD-1 group is limited, it provides preliminary evidences that comparing first-line anti-PD-1 monotherapy versus standard chemotherapy is a “trial-worthy” approach.

Interestingly, we observed similar CR rates between GP chemotherapy and anti-PD-1 + chemotherapy. Considering that anti-PD-1 monotherapy only achieved limited CR rate (~ 0–2%), it might suggest that the addition of anti-PD-1 agents may not be adequate enough to increase complete elimination of tumor cells (CR rate), though this combination therapy can still substantially improve tumor killing efficacy (PR rate). Likewise, a phase 2 trial by Chia and colleagues [12] reported a similar CR rate (8.6%) and a relatively higher PR rate (62.9%) in treatment-naive RM-NPC receiving chemotherapy followed by EBV-specific cytotoxic T lymphocytes (CTLs), compared to that of GP chemotherapy (Table 2). These results implicate that the combination of immunotherapy agents (either anti-PD-1 or EBV-CTLs) with chemotherapy may not achieve synergic effects (similar CR rates) in NPC patients; however, the substantially increased PR rate may still translate into patient survival benefits. Our findings need to be verified in ongoing/future prospective randomized trials evaluating the combination of immunotherapy and chemotherapy versus chemotherapy alone.

**Table 2:** Summary of characteristics and clinical endpoints of the Singapore trial evaluating the combination of chemotherapy and adoptive T-cell therapy in NPC

Items	Singapore trial
Characteristics	
Phase	2
Key eligibility criteria	Treatment-naïve recurrent/metastatic EBV-positive NPC
Experimental regimen	Chemotherapy+EBV-CTLs: Gemcitabine 1000 mg/m^2^, and carboplatin (AUC 2) (days 1, 8 and 15) q4wks for 4 cycles, followed by EBV-CTL 1 × 10^8^ cells/m^2^ on weeks 0, 2, 8, 16, 24, and 32
Sample size	35
Clinical endpoints	
Median follow-up, months	29.9
ORR (%)	71.5
CR (%)	8.6
PR (%)	62.9
SD (%)	28.6
PD (%)	0
All grade AEs (%)	NR
Grade 3–5 AEs (%)	NR

*Abbreviations*: *AUC* area under the curve, *CR* complete response, *EBV* Epstein-Barr virus, *EBV-CTLs* EBV-specific cytotoxic T lymphocytes, *NPC* nasopharyngeal carcinoma, *NR* not reported, *ORR* objective response rate, *q4wks* every 4 weeks, *PD* progressive disease, *PR* partial response, *SD* stable disease

One major challenge of immunotherapy remains that it only benefits small subsets of patients. Prior trial data [6, 9] showed a numerically higher ORR in patients with PD-L1–positive RM-NPC than in those with PD-L1–negative tumors; and high-PD-L1 expression was associated with better survival outcomes in both NPC and other head and neck cancer [13, 14]. We further performed pooled analysis to increase statistical power. Unfortunately, a significant margin was still not reached; the possible reason would be limited sample size. Moreover, PD-L1 expression alone may not be the only determinant of treatment benefits; it might be contingent on other factors in the tumor microenvironment that are yet to be identified. Future studies are warranted to identify reliable biomarkers for tailoring anti-PD-1 therapies. Improvement in treatment efficacy can also be achieved by breakthroughs in combination therapy. It is shown that camrelizumab+GP combination approach achieved a remarkable ORR in first-line treatment. Additionally, previous exposure to ipilimumab significantly improved antitumor activity of camrelizumab [8]. Going forward, these preliminary findings construct a road-map for the design of future trials to assess the efficacy of immuno-oncology cocktail and/or dual inhibition of immune checkpoints approaches in NPC. Results from relevant ongoing trials (e.g. NCT03581786, NCT03707509, NCT03097939) are eagerly awaited.

One major limitation of this study is that all trials were in phase 1/2, therefore long-term survival data are still lacking and the sample sizes were limited, especially for first-line nivolumab (*n* = 5) and the combination therapy group (*n* = 23). Our findings need to be verified in future large-scale, head-to-head, phase 3 trials. Secondly, the results of subgroup analyses regarding PD-L1 expression level should be interpreted with cautions, in view of the different immunohistochemical assays used [5, 6, 9].

## Conclusions

Our study comprehensively compares the safety profile and efficacy of anti-PD-1 monotherapy, chemotherapy and their combination in RM-NPC, which provides important evidence for the design of future trials and clinical management with respect to anti-PD-1 therapy.

## Data Availability

The datasets used and/or analyzed during the current study are available from the corresponding author on reasonable request.

## Abbreviations

AE: Adverse event
CI: Confidence interval
CR: Complete response
CTL: Cytotoxic T lymphocytes
ESMO: European Society for Medical Oncology
GP: Gemcitabine plus cisplatin
NPC: Nasopharyngeal carcinoma
OR: Odds ratio
ORR: Objective response rate
PD-L1: Programmed death-ligand 1
PR: Partial response
RM-NPC: Recurrent or metastatic NPC
